# Large-Area Fabrication of Laser-Induced Periodic Surface Structures on Fused Silica Using Thin Gold Layers

**DOI:** 10.3390/nano10061187

**Published:** 2020-06-18

**Authors:** Clemens Kunz, Sebastian Engel, Frank A. Müller, Stephan Gräf

**Affiliations:** Otto Schott Institute of Materials Research (OSIM), Friedrich Schiller University Jena, Löbdergraben 32, 07743 Jena, Germany; clemens.kunz@uni-jena.de (C.K.); sebastian.engel@uni-jena.de (S.E.); frank.mueller@uni-jena.de (F.A.M.)

**Keywords:** nanostructuring, femtosecond laser, laser-induced periodic surface structures, thin gold layer, transmission, wettability, silanization, functional surface properties

## Abstract

Despite intensive research activities in the field of laser-induced periodic surface structures (LIPSS), the large-area nanostructuring of glasses is still a challenging problem, which is mainly caused by the strongly non-linear absorption of the laser radiation by the dielectric material. Therefore, most investigations are limited to single-spot experiments on different types of glasses. Here, we report the homogeneous generation of LIPSS on large-area surfaces of fused silica using thin gold layers and a fs-laser with a wavelength *λ* = 1025 nm, a pulse duration *τ* = 300 fs, and a repetition frequency *f*_rep_ = 100 kHz as radiation source. For this purpose, single-spot experiments are performed to study the LIPSS formation process as a function of laser parameters and gold layer thickness. Based on these results, the generation of large-area homogenous LIPSS pattern was investigated by unidirectional scanning of the fs-laser beam across the sample surface using different line spacing. The nanostructures are characterized by a spatial period of about 360 nm and a modulation depth of around 160 nm. Chemical surface analysis by Raman spectroscopy confirms a complete ablation of the gold film by the fs-laser irradiation. The characterization of the functional properties shows an increased transmission of the nanostructured samples accompanied by a noticeable change in the wetting properties, which can be additionally modified within a wide range by silanization. The presented approach enables the reproducible LIPSS-based laser direct-writing of sub-wavelength nanostructures on glasses and thus provides a versatile and flexible tool for novel applications in the fields of optics, microfluidics, and biomaterials.

## 1. Introduction

The advantageous mechanical, physical, and chemical properties of technical and optical glasses, such as borosilicate glass, soda-lime-silicate glass, and fused silica, make them the material of choice for a large variety of applications in the fields of optics, microfluidics, photovoltaics, and biomaterials. The optimal performance of the utilized materials often requires a well-defined adjustment of the surface properties. These include the tribological behavior, specific wetting states, the optical response, as well as the behavior of the surface in contact with living cells. In addition to a modified surface chemistry and the usage of functional coatings, the surface properties are influenced by a well-defined adjustment of the surface topography. In this context, nature developed numerous outstanding skills and structures. These include the probably most known example of the lotus leaf with its superhydrophobic and self-cleaning properties [1] and the antireflective properties of the moth’s eye [2], to name only a few examples. In order to make these functional principles also available to technical applications, processes are required that allow the generation of the appropriate structures on the surface of different types of materials. In this context, lasers emerged as a versatile and flexible tool. In particular, the generation of so-called laser-induced periodic surface structures (LIPSS) using ultra-short pulse lasers (fs-lasers) attracted increasing importance in recent years [3,4,5]. LIPSS result from the irradiation of the surface with linearly polarized fs-laser radiation close to the ablation threshold and they can be characterized as a modulation of the surface topography having spatial periods *Λ* typically smaller than the utilized fs-laser wavelength *λ* [6].

Available studies on LIPSS formation on glasses are mainly limited to single-spot experiments demonstrating that different types of LIPSS can be generated in dependence on the fs-laser peak fluence *F* and the number of pulses *N* [7,8,9,10,11,12,13]. According to *Λ*, they are divided into high-spatial frequency LIPSS (HSFL) and low-spatial frequency LIPSS (LSFL). While LSFL on glasses are characterized by *Λ* ~ *λ*/*n*, with *n* being the refractive index of the material, and an orientation parallel to the electrical field (*E*-field) vector, HSFL exhibit remarkably smaller periods (*Λ* << *λ*) and an orientation perpendicular to *E* [8,9]. For theoretical approaches to explain the formation of both LIPSS types, the reader is referred to the literature [3]. Briefly, the most-widely accepted theory to explain LSFL formation is based on interference of the incident laser radiation with surface electromagnetic waves that are generated by scattering at the rough surface [14]. As an alternative approach, self-organization of the irradiated material was used to explain the formation of HSFL and LSFL [7,15]. In this framework, LIPSS formation is described based on processes occurring during the relaxation of the surface from a laser-induced state of thermodynamic instability. Rudenko et al. recently performed numerical studies for fused silica suggesting that the formation of both LIPSS types is driven by the interference of the incident laser field with the scattered (non-radiative) near-field (HSFL) and the (radiative) far-field (LSFL), respectively, below the surface [16]. This requires the presence of initial inhomogeneities, electron defects, or scattering centers.

Despite the numerous investigations and promising results, only a few studies focused on a line-like or even area-like generation of LIPSS on glasses by a relative movement (scanning) of the laser beam [17,18,19]. Here, Papadopoulos et al. should be mentioned, who systematically investigated the fabrication of large areas on fused silica containing pillar-like laser-induced nanostructures by means of circularly polarized laser radiation [19]. The low number of studies is caused by some challenges during LIPSS formation on glasses, which are mainly related to their amorphous chemical structure and the relatively large band gap energy, when compared to metals and semiconductors. The latter results in strongly non-linear (multi-photon) absorption processes at the typical laser wavelengths. Consequently, glasses are more sensitive to changing irradiation properties, defects, and incubation effects.

Alternative approaches of large-area structuring are therefore based on dot-structures, hierarchical surface structures, and single-spot matrixes [20,21,22,23]. Furthermore, the application of metallic and semiconducting thin films, deposited on the substrate surface prior to laser structuring, was demonstrated to be advantageous for the formation of LIPSS on large bandgap materials including sapphire [24,25], glasses [26,27], and silicon [28]. Here, we report on the use of thin gold layers (≤300 nm) to enable the fabrication of homogenous, large-area LIPSS pattern on fused silica substrates. The investigations focus on HSFL as the spatial periods *Λ* of these structures are typically in the order of 200–400 nm, which is why they are expected to be advantageous for optical applications in the visible spectral range. Based on a detailed single-spot analysis of the formation process as a function of the laser parameters and the layer thickness, the large-area fabrication of LIPSS was studied by means of an unidirectional scanning of the focused fs-laser beam across the substrate surface. The nanostructured surfaces were subsequently analyzed with regard to their topography and surface chemistry and characterized with respect to selected functional properties that might open up new applications in fields such as optics, microfluidics, and photovoltaics.

## 2. Materials and Methods

### 2.1. Sample Preparation

**Figure 1:**
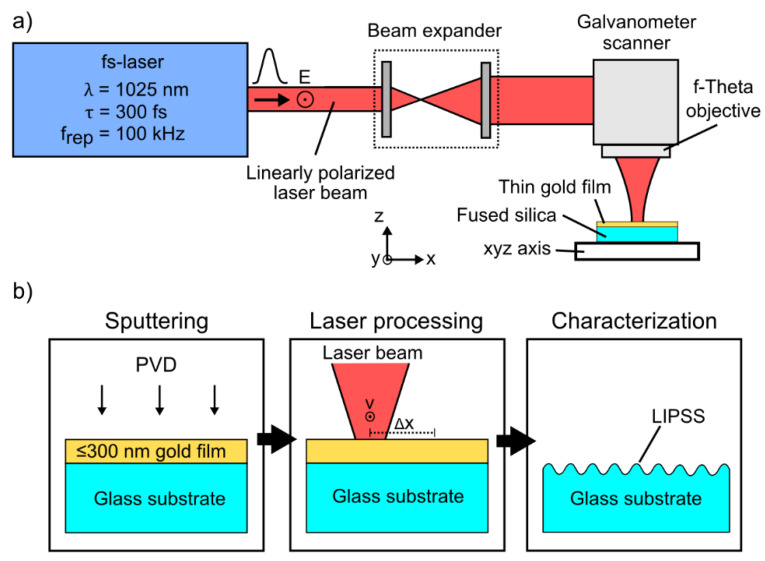
(**a**) Scheme of the experimental setup used for the generation of laser-induced periodic surface structures (LIPSS) on fused silica and (**b**) processing steps required for the large-area structuring with LIPSS.

Samples of commercial fused silica (GVB, Herzogenrath, Germany) with a size of (20 × 10 × 1 mm^3^) were used as substrate material (Figure 1b). Thin layers of gold with a thickness ≤300 nm were deposited on the substrate surface by sputtering (Sputter Coater S150B, Edwards, Irvine, CA, USA). The corresponding film thickness was determined using a white-light interference microscope (CCI HD, Taylor Hobson, Leicester, UK) equipped with a 50× objective. All samples were ultrasonically cleaned in acetone and isopropanol before sputtering and after fs-laser processing (Figure 1b). The wettability of the sample surfaces before and after fs-laser irradiation was modified by silanization with trichloro(1H,1H,2H,2H-perfluorooctyl)silane (Alfa Aesar, Haverhill, MA, USA). For this purpose, the samples were placed in a desiccator close to a 100 µL drop of the silane, which was deposited on the surface by gas phase condensation using a vacuum pump for 15 min and further storage of the samples for 15 min in the desiccator. After the deposition, the samples were stored for 2 h at 75 °C in a furnace.

### 2.2. Laser Processing

A diode pumped Yb:KYW thin disc fs-laser (JenLas D2.fs, Jenoptik, Jena, Germany) emitting linearly polarized fs-laser pulses (pulse duration *τ* = 300 fs, repetition frequency *f*_rep_ = 100 kHz) at a central wavelength *λ* = 1025 nm was used as radiation source (Figure 1a). The fs-laser beam was focused by a galvanometer scanner (IntelliScan14, Scanlab, Puchheim, Germany) including a f-Theta objective (JENar, Jenoptik, Jena, Germany) with a focal length *f*_L_ = 100 mm. The resulting focal spot diameter of the Gaussian beam (1/*e*^2^ intensity) on the substrate surface was determined to be 2*w*_f_ ~ (22 ± 0.5) µm using the method proposed by Liu [29]. Single-spot experiments were used to evaluate the formation process of LIPSS as a function of the gold layer thickness *t*. For this purpose, the substrate surface was irradiated at ambient air atmosphere by *N* = 5 successive pulses with single-pulse energies *E*_imp_ ≤ 31 µJ resulting in a maximum fs-laser peak fluence *F* = 2·*E*_imp_/(π·*w*_f_^2^) = 16.3 J/cm^2^. In this context, the threshold fluence of LSFL formation (*F*_LSFL_) was determined, as it indicates the transition from HSFL to LSFL and thus clearly identifies the fluence range suitable for HSFL formation. Large surface areas structured with LIPSS were realized by scanning the focused fs-laser beam (*F* = 4.5 J/cm^2^) with a velocity *v* = 0.15 m/s unidirectionally across the sample surface using different values of the line spacing Δ*x* and thin gold layers. In this context, the effective number of laser pulses *N* per focal spot area is given by *N*_eff_ = (π·*w*_f_^2^·*f*_rep_)/(*v*·Δ*x*).

### 2.3. Characterization

The morphology of the sample surfaces was evaluated by scanning electron microscopy (SEM) (Sigma VP, Zeiss, Oberkochen, Germany) at an accelerating voltage of 5 kV using the secondary electron detector. The surface topography including the modulation depth *h* of the LIPSS and the resulting roughness factor *r* was characterized by atomic force microscopy (AFM) (NanoWizard 4, JPK, Berlin, Germany) using a silicon tip (SNL-B, Bruker, Billerica, MA, USA) with a spring constant of 0.23 N/m and a resonant frequency of 23 kHz in contact mode. The spatial periods of the LIPSS were quantified by 2D-Fast-Fourier transform (2D-FFT) analyses of the SEM micrographs and verified by AFM. The chemical composition of the sample surfaces before and after fs-laser irradiation was analyzed by Raman spectroscopy (Senterra, Bruker, Billerica, MA, USA). The measurements were performed at a wavelength of 785 nm with an intensity of 100 mW and a 100× objective in a wavenumber range from 200 to 1800 cm^−1^ with a resolution of 3–5 cm^−1^. The influence of the LIPSS formation on the functional properties was evaluated in terms of the optical properties of the sample surfaces and their wettability behavior. For this purpose, the transmission of the samples was measured in a wavelength range from 400 to 1000 nm using an integrating sphere (IS236A-4, Thorlabs, Newton, NJ, USA) and a halogen–deuterium lamp (Tidas, J&M Analytik, Essingen, Germany) as radiation source. The integration time was set to 1 s and the signal was accumulated over 30 single measurements. The wettability of the surfaces with distilled water was analyzed by contact angle (CA) measurements (Drop-shape analyzer 10 Mk2, Krüss, Hamburg, Germany) at a minimum droplet volume of 4 µL in the sessile drop mode.

## 3. Results and Discussion

### 3.1. Single-Spot Investigation

**Figure 2:**
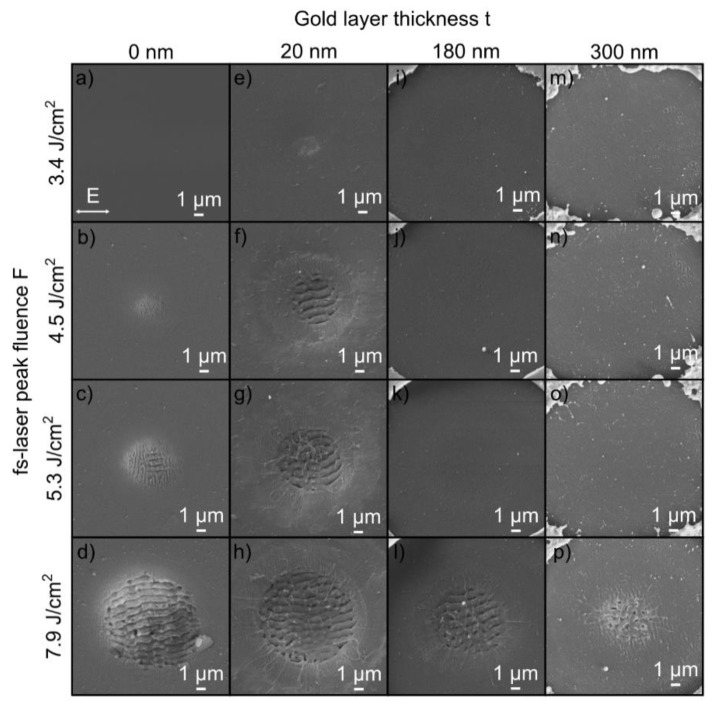
SEM micrographs of the fused silica surface upon the irradiation with *N* = 5 fs-laser pulses as a function of fs-laser peak fluence *F* and gold layer thickness *t*: (**a**–**d**) uncoated fused silica surface, (**e**–**h**) *t* = 20 nm, (**i**–**l**) *t* = 180 nm and (**m**–**p**) *t* = 300 nm. The direction of the linear beam polarization (*E*-field vector) is illustrated in (**a**).

In order to evaluate the impact of the thin gold layer on LIPSS formation, the layer thickness *t* was varied from 20 to 300 nm and compared to the LIPSS formation on the initial fused silica surface as reference. Figure 2 shows ablation spots irradiated with *N* = 5 fs-laser pulses as a function of the fs-laser peak fluence *F* (top to bottom) and the gold layer thickness *t* (left to right). Without a gold layer (Figure 2a–d), the typical dependence of fused silica on the fs-laser peak fluence can be observed in the focal spot area [8,9]. At a specific threshold, a transition from HSFL (Figure 2b) generated at low fluences (*F* = 4.5 J/cm^2^) to LSFL (Figure 2c) occurs. For the initial fused silica substrate, the threshold of LSFL formation was determined to be *F*_LSFL_ = 5.1 J/cm^2^ (Figure) at the given processing conditions in line with our former investigations [9]. This transition is characterized by a change in the orientation of the LIPSS from perpendicular (HSFL) to parallel (LSFL) with respect to the direction of the linear beam polarization (see Figure 2a). At the highest fluence *F* = 7.9 J/cm^2^ (Figure 2d), the ablation spot is characterized by well-pronounced, homogeneous LSFL in the intense center of the Gaussian beam profile, which are surrounded by an annular region containing HSFL. These experimental observations correlate very well with the numerical studies on LIPSS formation on fused silica reported by study, the Rudenko et al. [16]. In the corresponding authors revealed the interference below the surface between the incident laser field and the scattered near-field (HSFL) and the far-field (LSFL), respectively, to be the fundamental mechanisms of formation. Both LIPSS types are therefore generated at different sub-surface regions. Consequently, LSFL require sufficient high fluences for their “exposure” by an increased ablation of the material surface.

**Figure 3:**
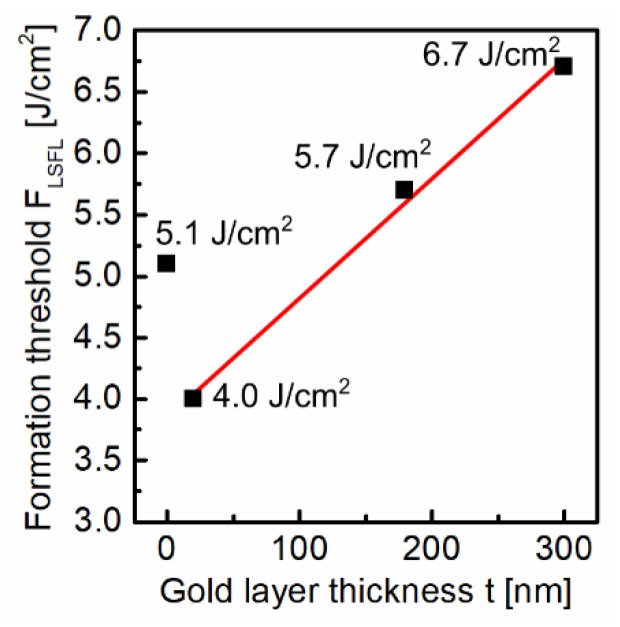
Threshold fluence *F*_LSFL_ for the formation of LSFL as a function of the gold layer thickness *t* determined from the corresponding SEM micrographs (see Figure 2) using the method proposed by Liu [29]. The red solid line guides the eye.

The ablation spots fabricated using the 20 nm thick gold layer (Figure 2e–h) reveal a shift of the fluence range of LIPSS formation towards lower values of *F*. This is indicated by the onset of HSFL formation already at *F* = 3.4 J/cm^2^. Furthermore, the comparison with the initial fused silica surface at *F* = 4.5 J/cm^2^ shows a significantly increased annular region containing HSFL as well as the formation of well-pronounced LSFL in the intensive center of the Gaussian beam profile. The corresponding value of the formation threshold was determined to be *F*_LSFL_ = 4.0 J/cm^2^, which is about 80% of the value of the uncoated fused silica surface (Figure 3). On the contrary, the SEM micrographs of the sample surfaces with a larger layer thickness show LIPSS formation only at the largest investigated fluence *F* = 7.9 J/cm^2^. Obviously, a thin gold layer leads to a lower formation threshold *F*_LSFL_ compared to the uncoated substrate, which increases almost linearly with increasing layer thickness *t* within the investigated thickness range (Figure 3).

In order to explain this impact of thin metallic films on LIPSS formation, the optical properties of the gold layer and its ablation behavior have to be discussed more detailed. At *λ* = 1025 nm, the complex refractive index of gold is *n* * = *n* + i*k* = 0.2277 + i6.4731 [30] corresponding to a complex dielectric permittivity *ε* = *n* * ^2^ = −41.85 + i2.95. The optical penetration depth *l*_α_ = *α* ^−1^ = λ/(4*πk*) can therefore be estimated to be *l*_α_ ~ 12 nm. According to Fresnel’s formulas, the reflectance at perpendicular incidence is about 0.98. The ablation of thin gold layers has already been investigated in detail using different pulse durations with a particular focus on the influence of the layer thickness *t* [31,32,33,34]. In the corresponding studies, the authors reported two different regimes upon fs-laser irradiation: For layer thicknesses below a characteristic penetration depth *L*_c_ of the pulse energy into the material, the ablation threshold linearly increases with increasing *t* and remains constant at its bulk value for *t* ≥ *L*_c_. During the fs-laser treatment (τ = 28 fs, λ = 793 nm) of gold films deposited on BK7 substrates, Krüger et al. determined *L*_c_ to be ~180 nm [32]. *L*_c_ is a measure for the heat penetration depth within the electron gas before electron–phonon relaxation occurs [32]. Therefore, it depends on the strength of electron-phonon coupling, which in the case of gold is relatively low [33]. Consequently, hot electrons excited by the deposited laser energy can penetrate deep into the material before an interaction with the lattice takes place, which is why *L*_c_ can noticeably exceed *l*_α_. As the energy transport across the metallic–dielectric interface can occur via electron–phonon and phonon–phonon coupling [35,36], the increased interfacial electron density resulting from the diffusion of the hot electrons leads to an increased interfacial energy transfer [24,28]. In contrast to the energy coupling to the uncoated glass surface, which is solely based on strongly non-linear multiphoton processes, this leads to a stronger, more uniform deposition of the energy to the fused silica substrate and thus to the significant reduction of the LIPSS formation threshold. This reduction of *F*_LSFL_ is more pronounced in the case of small layer thicknesses (*t* = 20 nm) in the order of *l*_α_. Plasmonic effects may also contribute to this process [37]. This aspect is emphasized by the fact that about 20% of the absorbed energy is still present after transmitting the 20 nm thick gold layer, which can therefore interact with the interfacial carriers. Furthermore, it should be noted that the increased coupling of the first pulse also influences subsequent processes such as accumulation and incubation occurring during multi-pulse irradiation. The observed increase in *F*_LSFL_ with increasing layer thickness is connected to the reported linear increase of the ablation threshold of the gold film and results from the reduced coupling and transmission of the incident laser radiation through the layer to the fused silica substrate (optical penetration depth) as the film thickness increases. Furthermore, thicker gold layers require more energy to ablate the respective layer [38]. Consequently, at larger film thickness, less laser energy is available on the fused silica surface for LIPSS formation.

Using metallic layers, the formation of LIPSS with significantly enhanced homogeneity was reported by several groups on different types of materials [24,25,28]. As the main reason, the authors assume the gold-layer-assisted homogeneous coupling field to be responsible, which reduces the influence of material properties (e.g., inhomogeneities and defects) and laser energy fluctuations. As a result, the first laser pulses generate a more homogenous modification of the material that acts as seeds for the formation of a well-pronounced LIPSS pattern by the subsequent laser pulses. This feedback mechanism includes various multi-pulse processes such as accumulation, incubation and grating-assisted coupling [3]. While a comparable impact on the LSFL was not observed within the present study, the annularly arranged, filigree HSFL fabricated with a 20 nm thin gold layer are more pronounced and more homogenous than on the uncoated substrate (Figure 2f–h).

### 3.2. Large-Area Fabrication of HSFL

**Figure 4:**
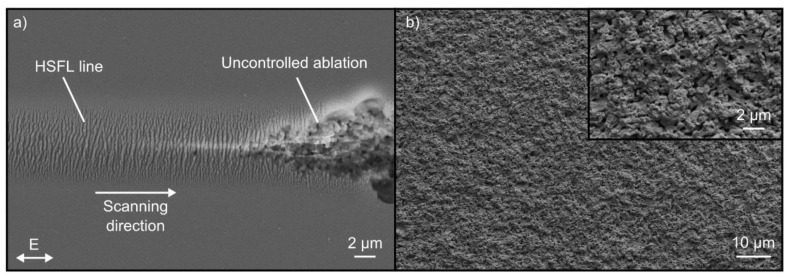
SEM micrographs of an uncoated fused silica surface upon fs-laser irradiation with *F* = 4.9 J/cm^2^: (**a**) high-spatial frequency LIPSS (HSFL) generated by a single line scan with *v* = 0.15 m/s; (**b**) Large-area structuring by unidirectional scanning with *v* = 0.15 m/s and Δ*x* = 4 µm. The direction of the *E*-field vector is indicated in (**a**).

Based on the fluence ranges evaluated in the single-spot experiments, the following investigations aim at the realization of large areas homogenously structured with HSFL. An important step is the generation of single scan lines, which will later be extended by an unidirectional scanning of the surface with a suitable line spacing Δ*x*. Figure 4a shows an example of a single scan line containing HSFL fabricated with *v* = 0.15 m/s and *F* = 4.9 J/cm^2^. In principle, the first part of the scan line reveals that the nanostructures generated within the focal spot can be written continuously on an uncoated fused silica surface. However, the SEM micrograph also emphasizes the typical problems occurring during LIPSS formation on glasses, which are caused by the strong influence of material inhomogeneities, surface contaminations, defects, and fluctuations of the pulse energy. As soon as the narrow process window is left, the structuring process switches to uncontrolled ablation, which leads to significant damage of the substrate surface. These problems are intensified when structuring large areas (Figure 4b). Here, the much smaller width of the ablated line (~8 µm) compared to the focal diameter (~22 µm) requires a certain overlap of the individual scan lines. The fs-laser fluence in the outer areas of the Gaussian profile is insufficient for the formation of HSFL, but large enough to alter the absorption properties by incubation effects, which might include the formation of self-trapped excitons and color centers [39,40,41,42]. The energy of a subsequent scan line then couples more strongly into the modified surface areas and also results in uncontrolled ablation. This results in an irregular roughness of the ablated surface instead of the intended HSFL. In order to reduce the influence of the versatile, non-controllable influencing parameters, the structuring process was systematically investigated using thin gold layers with a thickness of 20 nm.

**Figure 5:**
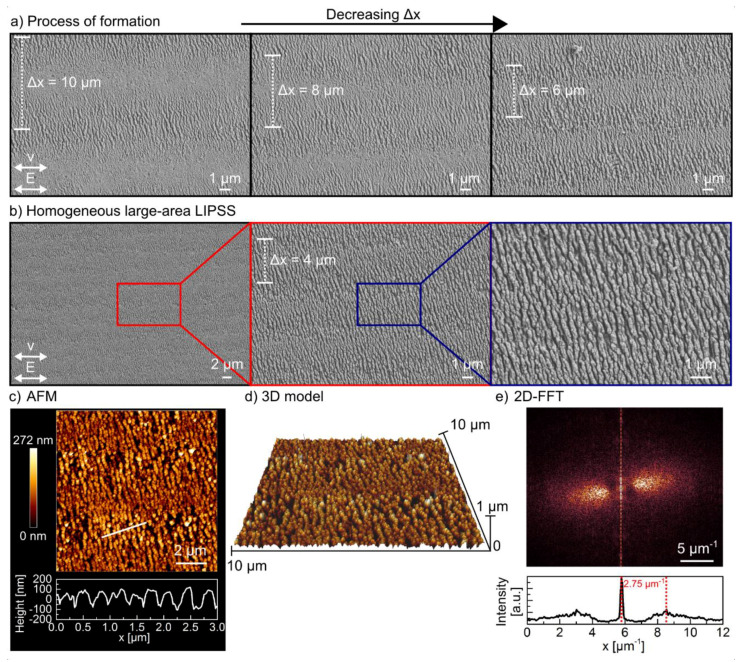
Large-area structuring of fused silica substrates homogeneously with HSFL using a 20 nm thin gold layer: (**a**) SEM micrographs of line scans performed with *v* = 0.15 m/s, *F* = 4.5 J/cm^2^, and different line spacing ∆*x* ranging from 10 to 6 µm; (**b**) SEM micrographs of different magnification showing a homogenous HSFL pattern fabricated with *v* = 0.15 m/s (*N* = 63), *F* = 4.5 J/cm^2^, and ∆*x* = 4 µm; (**c**,**d**) atomic force microscopy (AFM) image, cross section; and 3D model of the HSFL shown in (**b**); (**e**) 2-D Fourier transform of the SEM micrograph shown in (**b**); Please note the direction of the *E*-field vector displayed in (**a**,**b**).

For this purpose, the focused fs-laser beam was scanned with *v* = 0.15 m/s line-wise across the fused silica surface using an fs-laser peak fluence of 4.5 J/cm^2^. The direction of the linear beam polarization was chosen parallel to *v*. Figure shows SEM micrographs of the substrate surface fabricated with line spacing Δ*x* decreasing from 10 to 4 µm. For Δ*x* = 10 µm, the single scan lines can clearly be distinguished. Here, a decrease of the spatial periods from the intensive center of the scan line in direction of the borders can be observed, which is related to the Gaussian intensity distribution (see also Figure 2c). Considering the ablation width of about 8 µm, Δ*x* = 4 µm corresponds to a seamless alignment of the individual scan tracks. In this case, the overlap of the lines (regarding the Gaussian spot diameter) is about 80%. The SEM micrograph shows a homogeneous pattern with a size of (47 × 32 µm^2^) containing HSFL with an orientation perpendicular to the *E*-field vector. From the AFM cross section measured along the white line (Figure 5c), the spatial period and the modulation depth were determined to be *Λ* = (328 ± 58 nm) and *h* = (163 ± 27 nm), respectively. The 2D-FFT of the SEM micrograph (Figure 5e) reveals a wider distribution of the spatial periods, as the analysis also considers deviations resulting from the fluence-dependency of the HSFL and the unidirectional scanning procedure. Consequently, HSFL with spatial periods ranging from 208 to 592 nm can be found on the fused silica surface. The most frequent period in the cross section of the 2D-FFT spectrum is about 2.75 cm^−1^, which corresponds to a spatial period of about 362 nm. The homogeneity can be improved by using top-hat beam profiles, but this was not the subject of the current work [43,44]. Nevertheless, the results demonstrate that a thin gold layer enables the reliable and reproducible fabrication of HSFL on large surfaces areas without damaging the fused silica substrate. As already explained in the single-spot experiments, we assume in accordance with the literature that the gold layer leads to a more homogeneous coupling field, which reduces the influence of material properties and laser energy fluctuations. Furthermore, due to its very high reflectance, the gold layer may act as a protective layer for those areas of the fluence profile that are insufficient for HSFL formation.

### 3.3. Characterization of Surface Properties

#### 3.3.1. Surface Chemistry

**Figure 6:**
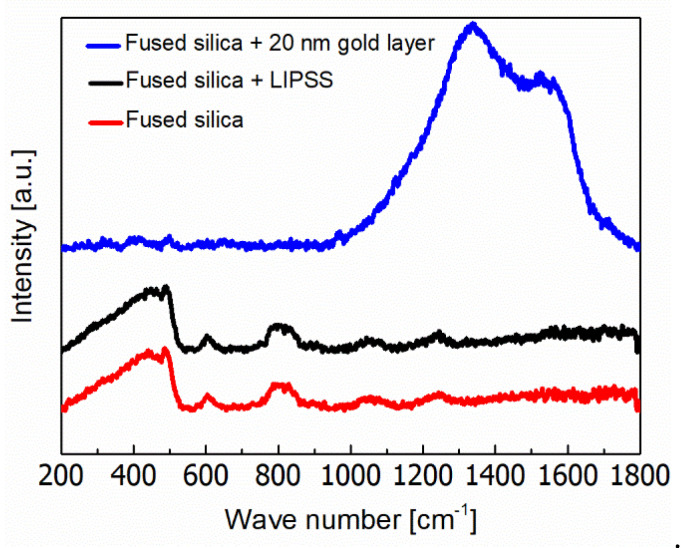
Raman spectrum of the initial, non-irradiated fused silica surface in comparison to fused silica surfaces covered with a thin gold layer before and after the formation of HSFL by fs-laser irradiation (*v* = 0.15 m/s, *F* = 4.5 J/cm^2^, Δ*x* = 4 µm) using a 20 nm thin gold layer. The spectra of initial fused silica and fused silica with LIPSS are magnified by a factor of 3.

Figure 6 shows the analysis of the surface chemistry by using Raman spectroscopy. The initial fused silica surface is characterized by a main broad peak at 435 cm^−1^ (w_1_), which corresponds to Si–O–Si bonds oscillating and bending in the SiO_4_ tetrahedrons [45,46,47]. Peaks detected at 488 and 604 cm^−1^ can be assigned to four- (D_1_) and three-membered (D_2_) rings of the SiO_4_ tetrahedrons, respectively [45,48]. At 800 cm^−1^, the spectrum reveals a peak of the symmetric stretching mode of the Si–O–Si (w_3_). Finally, peaks observed at 1078 and 1250 cm^−1^ correspond to the Si–O–Si asymmetric stretching mode (w_4_) with transversal and longitudinal optical modes, respectively [22]. After sputtering of a 20 nm thin gold layer, the spectrum is dominated by a broad peak consisting of two maxima at 1337 and 1530 cm^−1^. Both are related to organic residues, which might be caused by cleaning agent residues (acetone, isopropanol) as well as by carbon compounds adsorbed from the surrounding atmosphere [49]. The remarkably increased Raman intensity can be explained by the surface enhanced Raman scattering (SERS) effect reported for gold [50]. After LIPSS formation, the Raman spectrum equals to the initial fused silica surface. This confirms an almost complete ablation of the gold layer during fs-laser irradiation and excludes an impact of possible gold residues on the functional surface properties. Furthermore, a significant laser-induced modification of the chemical surface composition caused by LIPSS formation can be excluded [22,48].

#### 3.3.2. Wettability

**Figure 7:**
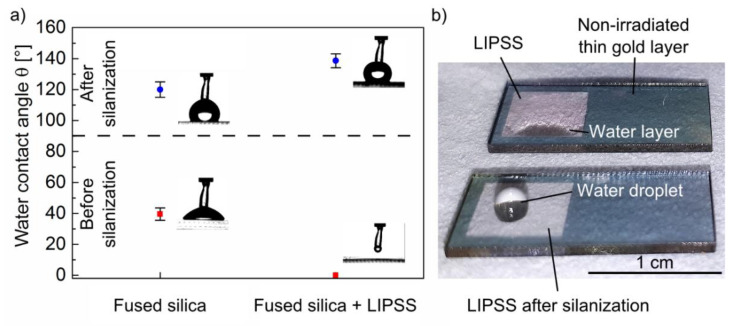
Contact angle analysis on fused silica with distilled water: (**a**) Wettability before and after silanization of non-irradiated substrates and surfaces homogeneously structured with HSFL. The inlets show a side-view image of the droplets during contact angle measurement; (**b**) Photographs of a droplet with a volume of 10 µL applied to the surface structured with HSFL without (top) and with (bottom) silanization (compare the corresponding inlets in (**a**)).

Figure 7 illustrates the analysis of the water contact angle *θ*_C_ of fused silica before and after fs-laser irradiation. The initial, non-irradiated fused silica surface is characterized by *θ*_C_ = (39.5 ± 4.0)°, which corresponds to a hydrophilic behavior of the flat surface, in line with literature values (Figure 7a) [21]. After fs-laser irradiation, the resulting homogeneous HSFL pattern leads to a significant reduction of *θ*_C_ to almost 0°, i.e., to a superhydrophilic wetting state. Consequently, a water droplet applied to the nanostructured surface spreads over the entire surface and forms a thin water film (top part of Figure 7b). The decreasing contact angle can be explained by the Wenzel model, which considers the impact of the surface roughness on the contact angle *θ* of an initially flat surface by the roughness factor *r* according to cos *θ*_W_ = *r* ∙ cos *θ* [51]. *r* represents the ratio of the real surface area to its horizontal projection. Using the Wenzel model, a theoretical value of *r* = 1.29 can therefore be calculated, which is in good agreement with *r* = 1.35 determined by AFM analysis for the fused silica surface covered with HSFL in this work. The small deviation can be attributed to fact that the Wenzel model only considers topographical aspects. In addition, several studies revealed a remarkable influence of the surface chemistry on the wetting behavior [52]. By means of a silanization of the material surface, the contact angle of hydrophilic materials can additionally be altered in a well-defined manner. Using trichloro(1H,1H,2H,2H-perfluorooctyl)silane, the contact angle of the non-irradiated fused silica surface was switched to hydrophobic with *θ* = (120.0 ± 5.0)°, which is caused by the hydrophobic fluoro-groups of the silane. As illustrated in Figure 7b, the silanization of the surfaces structured with HSFL led to a further increase of the hydrophobicity with a contact angle of *θ* = (139.0 ± 5.0)°. The comparison of the silanized samples before and after fs-laser irradiation directly reveals the influence of the LIPSS and their topography and therefore confirms that a monolayer was deposited during silanization. However, it has to be noted that a superhydrophobic behavior with contact angles exceeding 150° cannot be achieved by HSFL due to the small modulation depth of the structures. According to Cassie–Baxter, such superhydrophobic surfaces would require air pockets provided by the surface topography below the water droplet [53]. Nevertheless, our experimental results demonstrate that the wettability of fused silica surfaces can be tailored in a wide range from superhydrophilic to strongly hydrophobic based on the formation of HSFL. As shown below, this is achieved without a negative impact on the transparency of the substrates.

#### 3.3.3. Optical Properties

**Figure 8:**
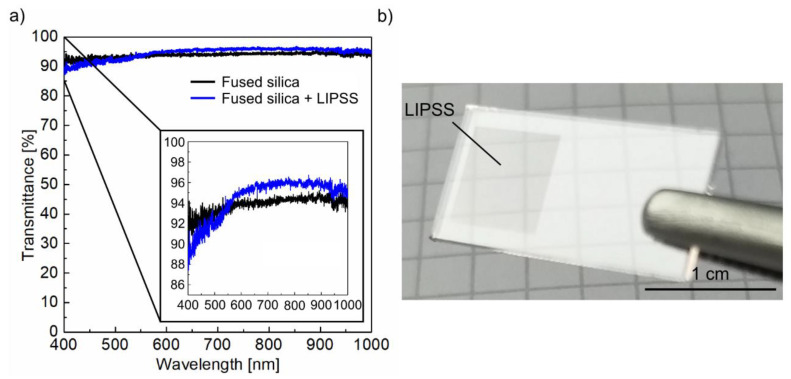
Optical characterization of fused silica samples before and after fs-laser irradiation: (**a**) transmittance measured using an integrating sphere and (**b**) photograph demonstrating the reduced reflectivity of the surface area homogenously structured with HSFL.

The analysis of the optical properties of the fused silica substrates in terms of their transmittance shows that the initial substrate is characterized by an almost constant value of about 93% in the investigated wavelength range from 400 to 1000 nm (Figure 8a). The remaining 7% to total transmittance is mainly caused by reflection at both substrate surfaces. For light irradiating a single-interface (air/fused silica) at normal incidence, the specular reflectance can be calculated using Fresnel’s formulas according to *R* = ((*n* − 1)/(*n* + 1))^2^, whereby *n* refers to refractive index of the transmitting material. The latter varies only slightly from *n* = 1.4696 at 400 nm incident wavelength to *n* = 1.4502 at 1000 nm in the case of fused silica [54]. This results in *R* ~ 3.5% for a single ideal interface in the investigated spectral range. It becomes evident from the inlet in Figure 8a that the fused silica substrate structured with HSFL on its top surface is characterized by an increased transmission when compared to the non-irradiated substrate for wavelengths exceeding 550 nm. At 800 nm wavelength, a maximum transmittance of about 96% was achieved. The increase in transmittance is based on a reduced reflectivity of the structured material surface, as also indicated in Figure 8b. This can be explained by the anti-reflective effect of the periodically structured material surface, which was discussed in several studies [55,56] and which is also well-known from natural examples such as the moth’s eye [2]. In addition to the specific grating profile and the depth of the generated structures, a key aspect is related to the period-to-wavelength ratio *Λ*/*λ*. For *Λ*/*λ* < 1, i.e., for wavelengths exceeding the spatial periods of the HSFL, the incident light is not diffracted. Instead, the incident radiation perceives effective optical properties at the material surface, which result from a structural mixing between incident and substrate material [55]. The interaction of light with such sub-*λ*-structures can be described with effective medium theories [55,56]. Below 550 nm, the wavelength equals to the periods measured for the HSFL (*Λ*/*λ* ~ 1). Here, diffraction of the incident radiation causes losses, which lead to a decrease in transmission of the substrate to ~88% at 400 nm. An improvement of the anti-reflective effect, especially by optimizing the structural geometry and structuring both substrate surfaces, will be the subject of future investigations.

## 4. Conclusions

The use of thin gold layers enables the reproducible generation of homogeneous LIPSS patterns on large surface areas of fused silica. A film thickness in the order of the optical penetration depth of gold results in a remarkable reduction of the threshold fluence of LIPSS formation. The nanostructuring of the fused silica surface leads to an increased transmission of the glass samples accompanied by a change in the wetting behavior of the substrate surfaces. The present study enables the utilization of LIPSS-based nanostructuring on glasses and might therefore open up new applications in the fields of optics, photovoltaics, anti-fogging, anti-icing, and biomaterials.
